# Symptoms, symptom relief and support in COVID-19 patients dying in hospitals during the first pandemic wave

**DOI:** 10.1186/s12904-021-00785-4

**Published:** 2021-07-01

**Authors:** Lisa Martinsson, Jonas Bergström, Christel Hedman, Peter Strang, Staffan Lundström

**Affiliations:** 1 Department of Radiation Sciences, Umeå University, SE-90187 Umeå, Sweden; 2 Palliative Care Unit, Stockholms Sjukhem Foundation, Stockholm, Sweden; 3 R & D Department, Stockholms Sjukhem Foundation, Stockholm, Sweden; 4 Department of Molecular Medicine and Surgery, Karolinska Institutet, Stockholm, Sweden; 5 Department of Oncology–Pathology, Karolinska Institutet, Stockholm, Sweden

**Keywords:** COVID-19, Pandemic, Palliative care, End-of-life care, Hospitals, Dementia

## Abstract

**Background:**

At the time of the first wave of the COVID-19 pandemic in Sweden, little was known about how effective our regular end-of-life care strategies would be for patients dying from COVID-19 in hospitals. The aim of the study was to describe and evaluate end-of-life care for patients dying from COVID-19 in hospitals in Sweden up until up until 12 November 2020.

**Methods:**

Data were collected from the Swedish Register of Palliative Care. Hospital deaths during 2020 for patients with COVID-19 were included and compared to a reference cohort of hospital patients who died during 2019. Logistic regression was used to compare the groups and to control for impact of sex, age and a diagnosis of dementia.

**Results:**

The COVID-19 group (1476 individuals) had a lower proportion of women and was older compared to the reference cohort (13,158 individuals), 81.8 versus 80.6 years (*p* < .001). Breathlessness was more commonly reported in the COVID-19 group compared to the reference cohort (72% vs 43%, *p* < .001). Furthermore, anxiety and delirium were more commonly and respiratory secretions, nausea and pain were less commonly reported during the last week in life in the COVID-19 group (*p* < .001 for all five symptoms). When present, complete relief of anxiety (*p* = .021), pain (*p* = .025) and respiratory secretions (*p* = .037) was more often achieved in the COVID-19 group. In the COVID-19 group, 57% had someone present at the time of death compared to 77% in the reference cohort (*p* < .001).

**Conclusions:**

The standard medical strategies for symptom relief and end-of-life care in hospitals seemed to be acceptable. Symptoms in COVID-19 deaths in hospitals were relieved as much as or even to a higher degree than in hospitals in 2019. Importantly, though, as a result of closing the hospitals to relatives and visitors, patients dying from COVID-19 more frequently died alone, and healthcare providers were not able to substitute for absent relatives.

## Introduction

The first cases of a new coronavirus disease, COVID-19, were reported in December 2019 in China [1, 2]. On 21 February 2020, nine European countries had reported a total of 47 cases [3]. COVID-19 is a new virus with new symptoms and trajectories [2, 4]. The narratives received from around the world picturing acute hospitals struggling with COVID-19 patients [5] caused the healthcare services to plan how to meet this pandemic on all different levels. It forced hospitals and caregivers to find new ways and treatments to provide the best possible care for these patients.

In the beginning of the pandemic palliative care guidelines were written, both in Sweden and worldwide, on how to provide patients dying from COVID-19 with good supportive care and how to alleviate symptoms [6–8], although little was known about what kind of problems to expect. At first glance, some symptoms may seem obvious in a lethal viral infection affecting the lungs, like breathlessness and respiratory secretions. However, other symptoms such as nausea, delirium, pain and anxiety are frequently seen near death in other conditions. It was not known how common those symptoms would be in hospitalised patients dying from COVID-19 or how well such symptoms could be alleviated. Little was known about the effect of our regular end-of-life care strategies, including drugs for symptom management in hospitals, for patients dying from COVID-19. However, emerging data show that the presentation of COVID-19 is diverse in both severity and symptoms.

The first wave in Sweden and worldwide was most intense during the spring 2020, with a new wave starting during the autumn and in Sweden in November 2020 [9, 10]. Early during the first wave, it was apparent that the initial global lack of personal protective gear [11] made it more difficult for healthcare professionals to be present bedside to the same extent that they normally are. Furthermore, there were restrictions on family visitation up until the time when the patient was immediately dying. This made it more difficult to provide optimal end-of-life care [5, 12–14]. The restrictions on family visitation were aimed at minimising spread of the disease but were also related to a shortage of personal protective gear.

Swedish healthcare is largely tax funded, and everyone should have equal access to healthcare services. The central government establishes principles and guidelines and sets the political agenda for health and medical care, but the actual care is decentralised to 21 regional councils that are expected to provide residents with good-quality health and medical care.

Nursing home residents, including many people with dementia, have been heavily affected by the pandemic. During the spring 2020 when deaths rates in nursing homes from COVID-19 were very high, there was a debate regarding old and fragile people with multiple diseases not always being offered hospital care when appropriate. Poloni et al. described in July 2020 that the combination of dementia and COVID-19 is associated with delirium [15] and with more unspecific and less prominent symptoms [16]. These facts indicate that people with dementia are a group of patients suffering from COVID-19 who require special attention, including in hospital settings.

## Aim

The aim was to study symptoms, symptom relief and support to patients dying from COVID-19 in hospitals, in comparison to a reference population (hospital deaths in 2019). Focus was on the occurrence and relief of symptoms during the last week of life, prescription of pro re nata (PRN) drugs against symptoms, administration of parenteral fluids during the last day, and the proportion of patients dying alone. A further aim was to control these factors for sex, age and a diagnosis of dementia, by the aid of logistic regression models.

## Methods

We used data from the Swedish Register of Palliative Care (SRPC), a national quality register focusing on quality of care during the last week in life, regardless of diagnosis, place of residence and level of care. Data are reported to the SRPC from healthcare staff after death of a patient, using a validated end-of-life questionnaire (ELQ) [17, 18]. Each year, around 60% of all deaths in Sweden are reported to the SRPC. The SRPC registers symptom data on breathlessness, anxiety, delirium, respiratory secretions, nausea, and pain. If the patient was indicated to have a symptom, symptom relieve was registered as complete, partial, or no relieve [17, 18]. The process that the SRPC uses for data collection has previously been described by our group [12].

The COVID-19 group consisted of all adults deceased in hospitals from the first reported death in March 2020 up until 12 November 2020 and reported to the SRPC as having a clinical COVID-19 infection. Both laboratory-verified and non-verified cases were included, mostly because laboratory testing was not possible in all cases in the beginning of the pandemic. The options regarding COVID-19 infection were “no infection”, “yes ongoing infection”, “yes suspected ongoing infection”, “earlier infection” and “unknown”. Those included in the COVID-19 group were: “yes ongoing infection”, “yes suspected ongoing infection”. All adults (≥ 18 years of age) deceased in hospitals between January 1st to December 31st 2019 and reported to the SRPC formed the reference cohort. Since most items in the SRPC used in this study, including symptoms, are not gathered for patients reported to have died unexpectedly, these patients were excluded from the analyses in both groups. Patients from all types of hospital wards (infection, geriatrics, internal medicine, surgery, etc.) except specialised palliative care wards (which cannot be distinguished from specialised palliative care wards outside of hospitals in the database) were included. Palliative care units were excluded because the majority of those units are outside hospitals in Sweden. In addition, the care in these units differs from usual hospital care which might affect the results.

As patients with dementia have been shown to present with different symptoms from COVID-19 compared to other patients, we adjusted the results for dementia in addition to sex and age.

### Statistical analyses

The COVID-19 group and the reference cohort were compared using t-test for comparison of means and chi-2 test for comparison of proportions. Logistic regression was used to control for the impact of age and to control for multiple independent variables. The following outcomes measured with the ELQ and collected from the SRPC were compared between the groups: occurrence of breakthrough of six symptoms during the last week of life (“Yes” vs “No”) and when present, relief of these six symptoms (breathlessness, anxiety, delirium, respiratory secretions, nausea and pain) during the last week of life (“Completely” vs “Partially” or “Not at all”); human presence at the time of death (close friend(s) and/or relative(s) vs staff or no one; and close friend(s) and/or relative(s) or staff vs no one); whether parenteral fluids/nutrition or enteral-tube feeding were administered during the last 24 h (“Yes” vs “No”); and whether PRN opioid against pain and PRN drugs against anxiety, nausea and respiratory secretions had been prescribed (“Yes” vs “No”). All items used in the analyses except age, sex and relief of symptoms when present could be answered with “Don’t know” in the ELQ, and these responses were excluded from the corresponding analysis.

Age was categorised into the following groups: 18–64, 65–74, 75–84, 85–94 and ≥ 95 years. In the analyses regarding relief of symptoms when present, only patients who were reported to have had that particular symptom were included. When analysing relief of symptoms when present, age was used as a continuous variable due to there being too few individuals eligible in each age category. Regarding those with dementia, symptom occurrence but not degree of relief of symptoms when present was analysed, because of too few patients having reported symptoms.

The Statistical Package for the Social Sciences (SPSS) version 26.0 (IBM Corp., Armonk, NY, USA) was used.

## Results

### Study population

A total of 10,748 hospital deaths in 2020 had been registered in the SRPC up until data retrieval (not including deaths that were registered as unexpected based on the disease trajectory). Of these, 1476 adult patients died with an ongoing COVID-19 infection according to the staff’s clinical assessment and were included in the study, and of these, 1247 were PCR-verified. The other cases were reported to have had a COVID-19 infection based on clinical presentation, out of whom 157 had tested negative, 47 had been tested but no answer was available and 13 had not been tested; for 12 it was unknown or not reported whether they had been tested.

A total of 13,158 adult hospital deaths during 2019 were identified in the SRPC (not including deaths that were registered as unexpected based on the disease trajectory), and these formed the reference cohort.

### Comparison between COVID-19 group and reference cohort

The COVID-19 group had a higher mean age (analysed with t test) and a lower proportion of women (analysed with chi2-test) compared to the reference cohort. The groups did not differ regarding the proportion of patients having dementia reported as a contributing cause of death (analysed with chi2-test) (Table 1).

**Table 1:** A comparison of patient characteristics between the COVID-19 group and the reference cohort. Analysed with t-test (age) and chi2-test (all other comparisons)

Characteristics	COVID-19 patients (***n*** = 1476)	Reference cohort from 2019 (***n*** = 13,158)	χ^**2**^	***P***-value
Age in years, mean (range)	81.8 (20–107)	80.6 (18–107)	-^c^	<.001
Female sex (%)	624/1476 (42%)	6303/13,158 (48%)	16.85	<.001
Dementia	103/1476 (7%)	828/13,158 (6%)	1.0	.31
**Breathlessness**^a^	953/1324 (72%)	5007/11,680 (43%)	406	<.001
Complete relief	286/953 (30%)	1428/5007 (29%)	0.9	.35
**Anxiety**^a^	825/1189 (69%)	5861/10,517 (56%)	81.3	<.001
Complete relief^b^	493/825 (60%)	3252/5861 (56%)	5.4	.021
**Delirium**^a^	433/1090 (40%)	3183/10,141 (31%)	31.3	<.001
Complete relief^b^	72/433 (17%)	482/3183 (15%)	0.6	.42
**Respiratory secretions**^a^	631/1357 (47%)	7098/12,397 (57%)	57.5	<.001
Complete relief^b^	223/631 (35%)	2222/7098 (31%)	4.4	.037
**Nausea**^a^	100/1022 (10%)	1472/10,214 (14%)	16.5	<.001
Complete relief^b^	40/100 (40%)	678/1472 (46%)	1.4	.24
**Pain**^a^	786/1283 (61%)	7996/11,934 (67%)	17.1	<.001
Complete relief^b^	538/786 (68%)	5153/7996 (64%)	5.0	.025
PRN opioid against pain^a^	1374/1458 (94%)	11,793/12,933 (91%)	15.7	<.001
PRN drug against anxiety^a^	1340/1453 (92%)	11,355/12,867 (88%)	20.5	<.001
PRN drug against nausea^a^	1122/1436 (78%)	9803/12,738 (77%)	1.0	.32
PRN drug against respiratory secretions^a^	1258/1448 (87%)	11,060/12,863 (86%)	0.9	.35
Close friend(s) and/or relative(s) present with or without staff at the time of death^a^	366/1419 (26%)	7114/12,736 (56%)	463	<.001
Someone present at the time of death^a^	805/1419 (57%)	9776/12,736 (77%)	271	<.001
Parenteral fluids/nutrition or enteral-tube feeding given during the last 24 h^a^	535/1433 (37%)	4406/12,782 (35%)	4.7	.031

^a^“*Don’t know*” was an optional answer for the questions on symptom occurrence, and these responses were excluded from the analysis *(for breathlessness n = 152, anxiety n = 287, delirium n = 386, respiratory secretions n = 119, nausea n = 454, pain n = 193 in the COVID-19 group and for breathlessness n = 1478, anxiety n = 2641, delirium n = 3017, respiratory secretions n = 761, nausea n = 2944, pain n = 1224 in the reference cohort)*. Therefore, numbers do not sum to group totals

^b^Alternatives for answers on symptom relief were “Completely”, “Partially” and “Not at all”

^c^The difference in mean age was calculated with t-test

*Abbreviation*: *PRN* = as needed.

Breathlessness was more commonly reported in the COVID-19 group compared to the reference cohort (72% vs 43%), χ^2^ (df = 1, *n* = 13,004) = 406, *p* < .001. Furthermore, anxiety (χ^2^ (df = 1, *n* = 11,706) = 81.3, *p* < .001), and delirium (χ^2^ (df = 1, *n* = 11,231) = 31.3, *p* < .001) were more commonly, and respiratory secretions (χ^2^ (df = 1, *n* = 13,754) = 57.5, *p* < .001), nausea (χ^2^ (df = 1, *n* = 11,236) = 16.5, *p* < .001) and pain (χ^2^ (df = 1, *n* = 13,217) = 17.1, *p* < .001) were less commonly, reported during the last week in life in the COVID-19 group. When present, complete relief of anxiety (χ^2^ (df = 1, *n* = 6686) = 5.4, *p* = .021), pain (χ^2^ (df = 1, *n* = 8782) = 5.0, *p* = .025) and respiratory secretions (χ^2^ (df = 1, *n* = 7729) = 4.4, *p* = .037) was more often reported in the COVID-19 group compared to the reference cohort. The proportion of patients who were completely relieved from breathlessness, delirium and nausea, when these symptoms were present, did not differ between the groups (Table 1).

Having close friend(s) and/or relative(s) present at the time of death (χ^2^ (df = 1, *n* = 14,155) = 463, *p* < .001), as well as having someone, including staff, present (χ^2^ (df = 1, *n* = 14,155) = 271, *p* < .001), was less common in the COVID-19 group. Parenteral fluids/nutrition or enteral-tube feeding was slightly more often given during the last 24 h in the COVID-19 group (χ^2^ (df = 1, *n* = 14,215) = 4.7, *p* = .031). Both individual prescriptions of injectable PRN opioids against pain (χ^2^ (df = 1, *n* = 14,391) = 15.7, *p* < .001) and PRN drugs against anxiety (χ^2^ (df = 1, *n* = 14,320) = 20.5, *p* < .001) were more common in the COVID-19 group, although the proportion of prescriptions was high also in the reference cohort. The prescriptions of PRN drugs against nausea and respiratory secretions did not differ between the groups (Table 1).

### Differences in symptom occurrence within the COVID-19 group

#### Chi-2 analyses and univariate logistic regression

In the chi-2 analysis, women more often had anxiety (χ^2^ (df = 1, *n* = 1189) = 4.7, *p* = .029) and nausea (χ^2^ (df = 1, *n* = 1022) = 7.6, *p* = .006) compared to men, and men more often had respiratory secretions (χ^2^ (df = 1, *n* = 1357) = 13.3, *p* < .001), within the COVID-19 group (Table 2). Occurrence of breathlessness, delirium and pain did not differ between men and women (Table 2).

**Table 2:** Sex differences within the COVID-19 group, not adjusted for age. Analysed with chi-2 test

Characteristics	Women	Men	χ^**2**^	***P***-value
**Breathlessness occurrence**^a^	403/555 (73%)	550/769 (72%)	0.2	.66
Breathlessness relief^b^	130/403 (32%)	156/550 (28%)	1.7	.20
**Anxiety occurrence**^a^	371/510 (73%)	454/679 (67%)	4.7	.029
Anxiety relief^b^	225/371 (61%)	268/454 (59%)	0.2	.64
**Delirium occurrence**^a^	176/455 (39%)	257/635 (41%)	0.4	.55
Delirium relief^b^	27/176 (15%)	45/257 (18%)	0.4	.55
**Respiratory secretions occurrence**^a^	232/570 (41%)	399/787 (51%)	13.3	<.001
Respiratory secretions relief^b^	82/232 (35%)	141/399 (35%)	0.00	.999
**Nausea occurrence**^a^	55/430 (13%)	45/592 (8%)	7.6	.006
Nausea relief^b^	21/55 (38%)	19/45 (42%)	0.2	.68
**Pain occurrence**^a^	345/548 (63%)	441/735 (60%)	1.2	.28
Pain relief^b^	236/345 (68%)	302/441 (69%)	0.001	.98
Close friend(s) and/or relative(s) present with or without staff at the time of death^a^	149/598 (25%)	217/821 (26%)	0.4	.52
Someone present at the time of death^a^	330/598 (55%)	475/821 (58%)	1.0	.32
Parenteral fluids/nutrition or enteral-tube feeding given during the last 24 hours	211/606 (35%)	324/827 (39%)	2.8	.092

^a^“*Don’t know*” was an optional answer for the questions on symptom occurrence, and these responses were excluded from the analysis. Therefore, numbers do not sum to group totals

^b^Alternatives for answers on symptom relief were “Completely”, “Partially” and “Not at all”

The occurrence of breathlessness was higher in the age groups of 65–74 (75%, OR = 2.69, 95% CI = 1.54–4.71, *p* = .001), 75–84 (76%, OR = 2.82, 95% CI = 1.72–4.62, *p* < .001) and 85–94 years (70%, OR = 2.05, 95% CI = 1.26–3.34, *p* = .004) compared to the oldest (≥95 years, occurrence 53%) in the univariate logistic regression model. There were no age differences seen in occurrence of anxiety, delirium, respiratory secretions, nausea or pain (Table 3).

**Table 3:** Age differences within the COVID-19 group, not adjusted for sex. Analysed with logistic regression

Characteristics	Age, years	Number of patients	OR	95% CI	***p*** value
Breathlessness occurrence^a^	18–64	42/63 (67%)	1.76	.88–3.50	.11
	65–74	138/183 (75%)	2.69	1.54–4.71	.001
	75–84	379/497 (76%)	2.82	1.72–4.62	<.001
	85–94	353/504 (70%)	2.05	1.26–3.34	.004
	≥95	41/77 (53%)	Ref		
Anxiety occurrence^a^	18–64	40/54 (74%)	1.60	.75–3.44	.23
	65–74	106/148 (72%)	1.41	.79–2.54	.25
	75–84	319/456 (70%)	1.30	.79–2.16	.30
	85–94	310/453 (68%)	1.21	.73–2.01	.45
	≥95	50/78 (64%)	Ref		
Delirium occurrence^a^	18–64	13/48 (27%)	.65	.29–1.46	.30
	65–74	53/149 (36%)	.97	.53–1.77	.91
	75–84	165/408 (40%)	1.19	.69–2.04	.53
	85–94	178/419 (43%)	1.29	.76–2.21	.35
	≥95	24/66 (36%)	Ref		
Respiratory secretions occurrence^a^	18–64	22/62 (36%)	.61	.31–1.19	.15
	65–74	83/185 (45%)	.90	.53–1.51	.68
	75–84	231/511 (45%)	.91	.57–1.45	.69
	85–94	256/517 (50%)	1.08	.68–1.72	.74
	≥95	39/82 (48%)	Ref		
Nausea occurrence^a^	18–64	6/43 (14%)	1.46	.44–4.88	.54
	65–74	19/135 (14%)	1.47	.56–3.90	.43
	75–84	39/378 (10%)	1.04	.42–2.56	.94
	85–94	30/406 (7%)	.72	.29–1.81	.48
	≥95	6/60 (10%)	Ref		
Pain occurrence^a^	18–64	34/64 (53%)	.71	.36–1.38	.31
	65–74	112/172 (65%)	1.17	.67–2.03	.59
	75–84	296/479 (62%)	1.01	.62–1.65	.97
	85–94	296/490 (60%)	.95	.58–1.56	.85
	≥95	48/78 (62%)	Ref		
Close friend(s) and/or relative(s) present with or without staff at the time of death^a^	18–64	20/71 (28%)	1.43	.68–3.01	.35
	65–74	71/203 (35%)	1.96	1.07–3.61	.030
	75–84	150/537 (28%)	1.41	.80–2.50	.23
	85–94	108/529 (20%)	.94	.53–1.67	.82
	≥95	17/79 (22%)	Ref		
Someone present at the time of death^a^	18–64	51/71 (72%)	2.62	1.33–.5.16	.006
	65–74	133/203 (66%)	1.95	1.15–3.30	.013
	75–84	312/537 (58%)	1.42	.89–2.28	.15
	85–94	270/529 (51%)	1.07	.67–1.72	.78
	≥95	39/79 (49%)	Ref		
Parenteral fluids/nutrition or enteral-tube feeding given during the last 24 hours^a^	18–64	37/70 (53%)	3.06	1.55–6.02	.001
	65–74	92/200 (46%)	2.32	1.32–4.08	.003
	75–84	213/545 (39%)	1.75	1.04–2.94	.034
	85–94	171/536 (32%)	1.28	.76–2.15	.36
	≥95	22/82 (27%)	Ref		

^a^“*Don’t know*” was an optional answer for the questions on symptom occurrence, and these responses were excluded from the analysis. Therefore, numbers do not sum to group totals

In the chi-2 analysis, patients with dementia were more often reported to have suffered from delirium (χ^2^ (df = 1, *n* = 1090) = 10.7, *p* = .001), and less often from anxiety (χ^2^ (df = 1, *n* = 1189) = 4.6, *p* = .033) and respiratory secretions (χ^2^ (df = 1, *n* = 1357) = 4.0, *p* = .044) compared to those without dementia (Table 4). These findings were confirmed in the multivariate regression model adjusting for age and sex (data not shown).

**Table 4:** Differences between patients with and without dementia in the COVID-19 group, not adjusted for sex or age. Analysed with chi-2 test

Characteristics	Dementia	No dementia	χ^**2**^	***P***-value
Breathlessness occurrence^a^	60/93 (65%)	893/1231 (73%)	2.8	.097
Anxiety occurrence^a^	47/80 (59%)	778/1109 (70%)	4.6	.033
Delirium occurrence^a^	47/83 (57%)	386/1007 (38%)	10.7	.001
Respiratory secretions occurrence^a^	36/98 (37%)	595/1259 (47%)	4.0	.044
Nausea occurrence^a^	6/80 (8%)	94/942 (10%)	0.5	.47
Pain occurrence^a^	59/92 (64%)	727/1191 (61%)	0.3	.56
Close friend(s) and/or relative(s) present with or without staff at the time of death^a^	23/99 (23%)	343/1320 (26%)	0.4	.55
Someone present at the time of death^a^	49/99 (50%)	756/1320 (57%)	2.3	.13
Parenteral fluids/nutrition or enteral-tube feeding given during the last 24 hours^a^	31/100 (31%)	504/1333 (38%)	1.8	.18

^a^“*Don’t know*” was an optional answer for the questions on symptom occurrence, and these responses were excluded from the analysis. Therefore, numbers do not sum to group totals

#### Multivariate analyses

The sex differences seen in the chi-2 analysis regarding anxiety, nauseas and respiratory secretions remained in the multivariate logistic regression model adjusting for age categories (Table 5) and in the multivariate logistic regression model adjusting for both age categories and dementia (data not shown).

**Table 5:** Sex differences (ref: women) in symptom occurrence in the COVID-19 group, adjusted for age categories (ref: oldest). Analysed with logistic regression

Characteristics	Sex differences (ref: women)			Age categories (ref: oldest)			
	OR	95% CI	*p* value	Age, years	OR	95% CI	*P*-value
Breathlessness occurrence^a^	.91	.71–1.16	.43	18–64	1.78	.89–3.54	.10
				65–74	2.76	1.57–4.86	<.001
				75–84	2.85	1.74–4.68	<.001
				85–94	2.07	1.27–3.37	.003
				≥95	Ref		
Anxiety occurrence^a^	.74	.57–.96	.020	18–64	1.66	.77–3.58	.20
				65–74	1.52	.84–2.73	.17
				75–84	1.34	.81–2.23	.25
				85–94	1.24	.75–2.06	.40
				≥95	Ref		
Delirium occurrence^a^	1.11	.86–1.42	.43	18–64	.64	.28–1.44	.28
				65–74	.94	.51–1.73	.85
				75–84	1.18	.69–2.02	.56
				85–94	1.29	.75–2.20	.37
				≥95	Ref		
Respiratory secretions occurrence^a^	1.53	1.23–1.90	<.001	18–64	.57	.30–1.13	.11
				65–74	.80	.47–1.35	.40
				75–84	.86	.53–1.37	.52
				85–94	1.03	.64–1.65	.90
				≥95	Ref		
Nausea occurrence^a^	.52	.34–.80	.003	18–64	1.68	.50–5.68	.40
				65–74	1.74	.65–4.67	.27
				75–84	1.11	.45–2.75	.83
				85–94	.76	.30–1.91	.56
				≥95	Ref		
Pain occurrence^a^	.87	.69–1.10	.24	18–64	.72	.37–1.41	.34
				65–74	1.20	.69–2.10	.52
				75–84	1.03	.63–1.68	.92
				85–94	.97	.59–1.58	.89
				≥95	Ref		

^a^“*Don’t know*” was an optional answer for the questions on symptom occurrence, and these responses were excluded from the analysis. Therefore, numbers do not sum to group totals

Occurrence of breathlessness was higher in the age groups of 65–74, 75–84 and 85–94 years compared to the oldest (≥95 years) in the multivariate logistic regression models adjusting for sex (Table 5), and adjusting for sex and dementia (data not shown). No other significant age differences regarding symptom occurrence were seen in these multivariate models.

### Differences in symptom relief within the COVID-19 group

There were no sex differences regarding relief of symptoms when present (Table 2). In univariate logistic regression using age as a continuous variable, relief of the following symptoms was more likely with increasing age: breathlessness (OR = 1.03, 95% CI = 1.01–1.04, *p* = .002), anxiety (OR = 1.03, 95% CI = 1.01–1.04, *p* = .001) and pain (OR = 1.02, 95% CI = 1.002–1.034, *p* = .026). These differences remained significant after adjusting for sex. Relief of delirium, respiratory secretions and nausea did not differ with age.

### Differences regarding someone present at time of death and parenteral fluids within the COVID-19 group

No sex differences were seen regarding the proportion of patients who died alone, the proportion of patients who had close friend(s) and/or relative(s) present at the time of death, or whether parenteral fluids/nutrition or enteral-tube feeding were administered during the last 24 h (Table 2). Furthermore, these outcomes did not differ between the groups with and without dementia within the COVID-19 group (Table 4).

Patients in the age group 65–74 years more often had close friend(s) and/or relative(s) present at the time of death compared to the oldest (OR = 1.96, 95% CI = 1.07–3.61, *p* = .030). The two youngest groups of 18–64 (OR = 2.62, 95% CI = 1.33–.5.16, *p* = .006) and 65–74 (OR = 1.95, 95% CI = 1.15–3.30, = .013) years were more likely to die with someone, that is, staff and/or close friend(s)/relative(s), present, compared to the oldest. The three youngest age groups, 18–64 (OR = 3.06, 95% CI = 1.55–6.02, *p* = .001), 65–74 (OR = 2.32, 95% CI = 1.32–4.08, *p* = .003) and 75–84 years (OR = 1.75, 95% CI = 1.04–2.94, *p* = .034), were more likely to receive parenteral fluids/nutrition or enteral-tube feeding during the last 24 h compared to the group 95 years and older (Table 3). These differences remained when adjusting for sex, and when adjusting for sex and a diagnosis of dementia (data not shown).

## Discussion

In this national register study on 1476 COVID-19-related deaths during the first pandemic wave at hospitals we found that breathlessness, anxiety and delirium were more common, while respiratory secretions, nausea and pain were less frequently seen during the last week in life compared to a reference cohort. When present, complete relief of anxiety, pain and respiratory secretions was more often achieved compared to the pre-pandemic population of dying patients in hospital. Several palliative care manuals for treating symptoms in patients with COVID-19 did foresee that respiratory secretions could be a common symptom and suggested treatment with antimuscarinic drugs [19–21].

We also found that a higher proportion of patients dying in hospitals due to COVID-19 died alone. The presence of next of kin was less common, and staff were not able to fully compensate for their absence, which was also seen in other countries [22]. This was probably due to lack of personal protective equipment as well as a general strain on healthcare personnel resources. Another possible explanation could be the difficulty of prognosticating dying and death in a new disease.

Younger patients in the COVID-19 group more often had someone present at the time of death. Since the SRPC data do not contain information about who received intensive care, we cannot say to what extent the level of care covariates with these results, but it is probable that patients who died in intensive care units had staff present to a much higher extent.

Interestingly, the proportion of people with dementia dying in hospitals did not differ between the COVID-19 group and the reference cohort. There has been a debate in Sweden regarding whether older people with multiple diseases, including dementia, residing in nursing homes were offered appropriate care for COVID-19 [23]. Our findings indicate that access to hospital care for people with dementia was similar to the access before the pandemic.

Within the group of patients dying from COVID-19 in hospitals, breathlessness was more common among younger patients. Furthermore, patients with dementia were more often reported to have suffered from delirium, and less often from anxiety and respiratory secretions compared to those without dementia. This is consistent with the findings by Martín-Sánchez et al. that described old age as a predictive factor for fewer respiratory and more unspecific symptoms from COVID-19 [24], and the findings by Poloni et al. that the combination of COVID-19 and dementia was associated with delirium [15]. In a recent study by our group, we showed that nursing home residents who died in hospital with COVID-19 were younger and also more often suffered from breathlessness and delirium during end of life compared to those who died with COVID-19 in their nursing home [25].

Furthermore, we also found that women who died from COVID-19 more often had anxiety and nausea the last week before death, while men more often suffered from respiratory secretions. While other studies also have found a symptom difference between women and men in mild to moderate COVID-19 [26, 27], it was somewhat surprising to find such measurable differences also for patients hospitalised for and subsequently deceased from COVID-19, after adjusting for age.

During the COVID-19 pandemic, assessment of prognosis and choice of whether invasive and potentially distressing treatments, such as mechanical ventilation, should be given or not, is especially demanding [28]. A person might be in a palliative stage, for example, due to a metastatic cancer disease with limited survival prospects in the long run. Still, the same person might have realistic chances to recover from COVID-19, which makes decisions more complicated in the face of a new disease with partly unknown trajectory, at least in the individual case [29]. However, in this study, we only selected patients whose death, according to hospital staff, was expected and foreseeable. Therefore, symptom control and comfort should have been main priorities.

Intravenous fluids administered during the last days of life may lead to complications such as pulmonary oedema and increased breathlessness [30]. Discontinuation of intravenous fluids such as artificial hydration and nutrition is generally recommended near end of life [1]. Slightly more patients dying from COVID-19 were prescribed parenteral administration of fluids and/or nasogastric feeding (37%) compared to patients dying in hospitals during 2019 (35%). This can possibly reflect prognostic uncertainty for COVID-19 and is perhaps also a product of the debate in Sweden during spring 2020 on whether elderly people with multiple diseases received appropriate care for COVID-19.

## Strengths and limitations

This study used data from a Swedish national quality register, the SRPC. The SRPC collects data about end-of-life care with an established method and contains data about more than 60% of all yearly deaths in Sweden since 2012. When the pandemic started, the SRPC added questions about COVID-19 and COVID-19 testing in their web-based end-of-life questionnaire, allowing data collection for this patient category early in the pandemic.

During the study period, PCR tests to verify the COVID-19 diagnosis were limited. Therefore, some of the patients included in this study had only a clinical diagnosis. Furthermore, for patients with multiple diseases reported to the SRPC, we cannot determine whether COVID-19 was the underlying or a contributing cause of death. We have not been able to access Swedish official cause of death data, as these are added from the National Board of Health and Welfare to the SRPC with a delay of more than 12 months. However, we have used a definition similar to that of the Public Health Agency (Folkhälsomyndigheten), which reports deaths for people with a reported COVID-19 diagnosis during the last 30 days as a death from COVID-19 [9].

## Conclusions

This national register study shows that although COVID-19 is a new disease with new symptoms and trajectories, the standard medical strategies for end-of-life care in Swedish hospitals seemed to work in an acceptable way during the first wave of the pandemic. Symptoms in COVID-19 deaths in hospitals in 2020 were relieved to a similar or even a higher degree than in hospitals in 2019.

Importantly, though, when the hospitals were closed to relatives and visitors, patients dying from COVID-19 more frequently died alone, and healthcare providers were not able to substitute for absent relatives.

## Data Availability

The dataset contains personally identifying information, such as personal identity numbers, and potentially identifying information, such as date of death, and therefore is subject to ethical and legal restrictions on public sharing. We cannot share the dataset, which is on an individual level, because it is not permitted according to the laws that apply in Sweden.

## Abbreviations

df: Degree of freedom
ELQ: End-of-life questionnaire
COVID-19: Coronavirus disease 2019
SRPC: Swedish Register of Palliative Care
PRN: Pro re nata (as needed)
PCR: Polymerase chain reaction
OR: Odds ratio
CI: Confidence interval
